# Micro-UFO (Untethered Floating Object): A Highly Accurate Microrobot Manipulation Technique

**DOI:** 10.3390/mi9030126

**Published:** 2018-03-14

**Authors:** Hüseyin Uvet, Ali Anil Demircali, Yusuf Kahraman, Rahmetullah Varol, Tunc Kose, Kadir Erkan

**Affiliations:** 1 Department of Mechatronics Engineering, Yildiz Technical University, Istanbul 34349, Turkey; anildemircali1@gmail.com (A.A.D.); rahmet@gmail.com (R.V.); tunc.kose89@gmail.com (T.K.); kerkan@yildiz.edu.tr (K.E.); 2 Science and Technology Research and Application Center, Yildiz Technical University, Istanbul 34349, Turkey; yusuf.kahraman@merklab.yildiz.edu.tr

**Keywords:** microrobots, untethered microrobotic manipulation, diamagnetic levitation

## Abstract

A new microrobot manipulation technique with high precision (nano level) positional accuracy to move in a liquid environment with diamagnetic levitation is presented. Untethered manipulation of microrobots by means of externally applied magnetic forces has been emerging as a promising field of research, particularly due to its potential for medical and biological applications. The purpose of the presented method is to eliminate friction force between the surface of the substrate and microrobot. In an effort to achieve high accuracy motion, required magnetic force for the levitation of the microrobot was determined by finite element method (FEM) simulations in COMSOL (version 5.3, COMSOL Inc., Stockholm, Sweden) and verified by experimental results. According to position of the lifter magnet, the levitation height of the microrobot in the liquid was found analytically, and compared with the experimental results head-to-head. The stable working range of the microrobot is between 30 µm to 330 µm, and it was confirmed in both simulations and experimental results. It can follow the given trajectory with high accuracy (<1 µm error avg.) at varied speeds and levitation heights. Due to the nano-level positioning accuracy, desired locomotion can be achieved in pre-specified trajectories (sinusoidal or circular). During its locomotion, phase difference between lifter magnet and carrier magnet has been observed, and relation with drag force effect has been discussed. Without using strong electromagnets or bulky permanent magnets, our manipulation approach can move the microrobot in three dimensions in a liquid environment.

## 1. Introduction

Microrobots are a promising method in biological and medical applications for a number of purposes, such as drug delivery, biopsy, marking, cell manipulation, micro particle transport, etc., with minimal damage to the desired site [1,2,3,4,5,6]. One of the most common application areas of microrobots is microfluidic systems [7]. In a microfluidic environment, flow characteristics and environmental effects, related with acting forces on a microrobot, change with decreasing dimensions. For example, decrease in Reynolds number causes a microfluidic environment to present laminar flow behavior. In the laminar flow environment, transfer and movement of micro-objects gets harder, since microfluidic system presents high viscous medium character [8]. In such laminar flow environment, different kinds of approaches can be used to transfer and move the micro-objects instead of using microrobots. These approaches can be counted as optical tweezers, thermal gradients, electrostatic forces, dielectrophoresis forces, and chemical concentration differences [9,10]. However, the use of the microrobots in such applications has more advantages over other applications when considering the spatial effects, the manipulation force to be applied, and the precision of micro/robot-object motion [2].

In biomedical applications such as single cell analysis, nanometer resolution is required to move and transfer some micro-objects [7,11]. Although microrobots have advantages when compared to other methods, there are still ongoing studies to develop microrobot’s precise movement capability. Viscosity of the medium in microfluidic chip and friction forces between microrobot and microfluidic substrate are the main challenges restricting precise motion and force output of the microrobot during manipulation [12]. For example, Bradley Nelson et al. were inspired by the artificial bacterial flagella (ABF) in the fabrication of their microrobot. This ferromagnetic microrobot has helical structure, and it can move by the action of rotating magnetic fields. In such a structure, rotational motion of tail allows high efficient motion with low magnetic fields [13]. In this type of microrobot design, helical structure appears to be a restriction, limiting the design of different robot geometries where there is a need for different microrobot designs in various microfluidic applications. Additionally, the microrobot does not conserve its position when the applied magnetic force is paused and the suspension of the microrobot is lost. Arai et al. have also been working on microrobot control with magnetic field effect using permanent magnets on the microrobot body, focusing on force output and position sensitivity of microrobot in their work [2]. Design of microrobots was modified in order to decrease friction force, leading to an improvement in accuracy of motion [3]. But there was still contact between substrate and microrobot as a limiting factor. Thus, ultrasonic vibrations were also introduced to microfluidic chips, in order to reduce surface friction forces [14]. All the effort was on reducing surface contact and having high force output to provide precise manipulation and effective cell manipulation. Ultrasonic vibrations were, however, also a factor that reveals a new problem that is the main reason of micro-objects’ vibrations and movement.

Recent studies by Metin Sitti and Arai show the use of magnetic levitation and acoustic levitation to provide precise positioning and high force output, respectively. The design of Metin Sitti’s study offers unstable motion control due to nonlinear distribution of electromagnetic field [15]. Unfortunately, it is hard to obtain time effective motion control. In their other work, a robot with 5 degrees of freedom (DOF) can be moved at higher speeds (>20 mm/s) by 8 electromagnets, although the orientation of the microrobot can be made with high positioning error of 2.83 mm [16]. In the study of Arai et al. ultrasonic external forces, which are applied for levitating microrobots, appeared once again as a restricting factor. The existence of the ultrasonic waves is still present in the liquid environment [17]. Also, Lucarini et al. investigated teleoperated and autonomous controls of a microrobot in a liquid environment. Even though they developed a robust control algorithm with high reproducibility to manipulate it at low speed of 2 mm/s, the mean error is as high as 250–300 µm. In addition, the effect of the control response was not mentioned in higher speeds [18]. Those efforts have shown that microrobot levitation studies had increasing demand to maintain better positioning accuracy and force output, whereas all those studies required constant energy consumption to drive piezo vibrator or run bulky electromagnets, in order to provide continuous suspension of the microrobot.

At this point, the diamagnetic levitation emerges as a powerful method for precise positioning of a microrobot with a suspension mechanism. With the aid of a permanent magnet embedded in the microrobot, which is positioned above a diamagnetic material, the microrobot can be levitated without an active control mechanism. In the studies on diamagnetic levitation, it is seen that, the dipole–dipole [19] interaction could be used by dividing the bismuth [20] and the permanent magnet into two halves, respectively. Unlike Pigot’s set-up, which works on a magnetic array, our system is in liquid, which is more suitable for lab-on-chip systems, and has higher control accuracy [20]. According to Profijt and his team, the levitation height is much lower than our proposed method [19]. To the best of our knowledge, the first diamagnetically floating microrobot application was developed by Ron Pelrine et al. [21,22]. In their work, impressive motion repeatability and speed results are demonstrated on the trajectory designed by the microrobot printed circuit board (PCB). However, in the proposed method, it is necessary to regulate the PCB and the 4-pole magnet for levitating the microrobot. Also, how to control the levitation height on the *z*-axis has not been specified. Despite the existence of a microrobot levitation technique, which moves horizontally very fast, there is no theoretical calculation, also minimum and maximum levitation ranges are not specified. In addition, the proposed levitation system is not in the liquid medium, so the potential for lab-on-a-chip applications have not been discussed. The current on the PCB affects the positioning accuracy of the passing path. In our case, the positioning resolution of the linear stage shows the same effect. The greater the PCB path width and the wider the path spacing, the lower the sensitivity is, because the uniformity of the distribution of the generated magnetic field is impaired.

Feng Ling and his team performed passive diamagnetic levitation using 4-pole magnets. However, the orbits can be 1 mm at most, and no motion sensitivities or control strategies are mentioned in their work [23]. Besides, in our method, the levitation height is controlled by one external ring-shaped permanent magnet, because we utilized pyrolytic graphite (PG) as a balancing force instead of a microrobot. Our robot is formed by SU8 and permanent magnet combination. Also, according to the position of the lifter magnet, microrobot 3D orientation can be provided. In our previous studies, the maximum and minimum microrobot operating points are also indicated by controlling the levitation height on the microrobot *z*-axis [24,25]. In those studies, we also show how to find optimum parameters in order to setup a diamagnetically levitated microrobot manipulation system. 

In this work, our proposed system provides a solution for three-dimensional motion control in liquid environments using single carrier magnet and lifter magnet. It does not require current control and can eliminate unwanted physical effects, such as heat and noise that can occur in other methods. According to similar approaches, size can be reduced by 1/4, and complex permanent magnet fusion methods are not needed. In the future, it will allow the construction of different polymer microrobots in which the buoyancy force can be used effectively. This study shows the analytical approach which includes theoretical, numerical, and experimental elements, including positioning accuracy, phase differences, and motion reaction, such as head tilting. The theoretically presented equations are solved numerically by FEM analysis. Design steps of microrobot levitation are demonstrated by multi-coupled physics analysis prior to the experimental stage. The proposed approach is experimentally confirmed. In addition, it is shown that complex trajectories can be tracked with submicron error. In this way, a high precision untethered microrobot manipulation technique has been developed without using bulky electromagnetic installations or extra sensory attachments to increase manipulation accuracy.

## 2. Mathematical Models

### 2.1. Schematic of Micro-UFO

**Figure 1:**
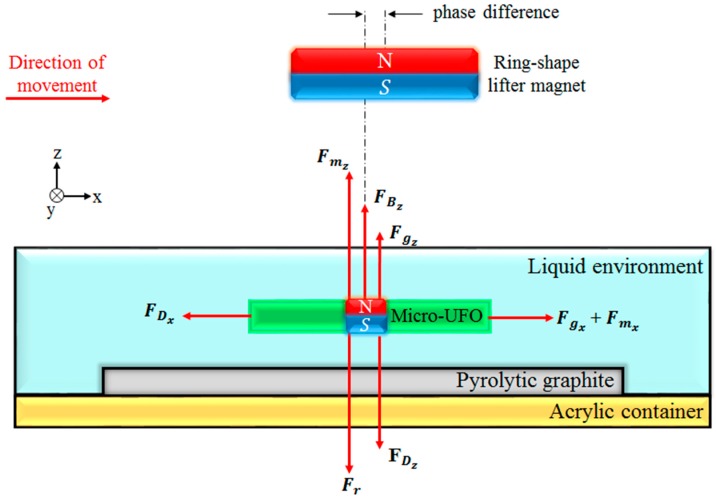
Shows the forces acting on the micro-untethered floating object (micro-UFO) during the *z*-axis levitation and lateral movement along the *x*-axis. The phase difference that occurs in the lateral movements is expressed as the distance between the center of the lifter and the carrier magnet.

By using diamagnetic force, precise levitation and contactless manipulation of the micro-untethered floating object (micro-UFO) can be performed [26,27,28]. According to Newton’s Second Law, the motion characteristics are determined by the forces which are exerted on the carrier magnet (shown in Figure 1). Firstly, system dynamics of the micro-UFO have been modelled to find out locomotion limits under the proposed condition. The point where the micro-UFO is suspended by the forces exerted on it in the liquid environment can be demonstrated by a mathematical model. Parameters used in the model are given in Table 1, and relevant forces are shown on the schematic (Figure 1).

**Figure 2:**
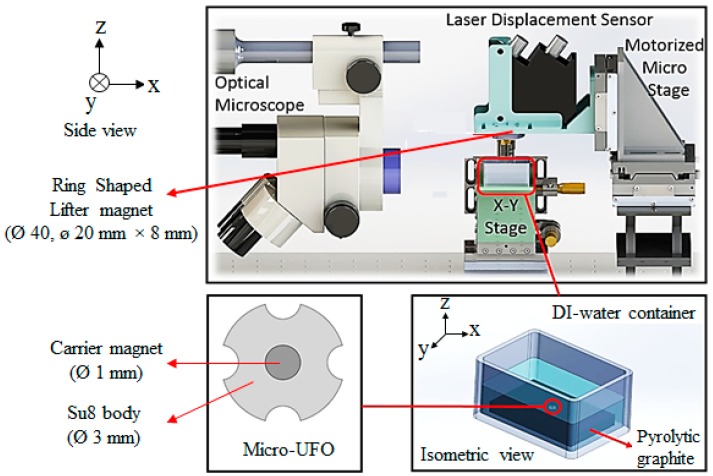
Shows the whole experimental setup with dimensions. The control of the motorized micro stage where lifter magnet is attached, is realized through a control interface programmed in Visual C#.Net platform (Microsoft Corporation, Redmond, WA, USA). Camera image is transferred to the same interface simultaneously. The data of laser displacement sensor is acquired through the sensors’ own interface.

In Figure 2, it is seen that the micro-UFO is positioned in a deionized (DI) water container with pyrolytic graphite on its surface. The ring-shaped magnet (lifter magnet) which generates the magnetic force required for levitation is positioned above the container. To achieve stable and micro-precise levitation, it is necessary to position the lifter magnet on the DI water container parallelly and rigidly. To do that, PI Micro Stage (M-126.PD2/20 mm × 20 mm × 20 mm, Physik Instrumente (PI) GmbH & Co. KG, Karlsruhe, Germany) with 3-axis linear micro/nano movement sensitivity was equipped. A manual micro-stage was used to position the DI water container parallel to the floor, and to move in 3 axes when it is necessary. Nano-sensitive laser distance sensor (optoNCDT-ILD2300-50, Micro-Epsilon, Raleigh, NC, USA) was preferred for instant measurement of levitation height within the system. At the same time, the microscope camera–lens system (Olympus SZX-7, Olympus Corporation, Tokyo, Japan and PointGrey GS3-U3, FLIR Integrated Imaging Solutions Inc., Richmond, BC, Canada) was positioned perpendicularly from the side profile to observe the mechanical contact of the micro-UFO with the pyrolytic graphite surface. Micro-UFO levitation was performed with an external ring shaped N48 grade “lifter magnet” and an N52 grade “carrier magnet” located at the SU-8 body center.

According to configuration of the experimental setup shown in Figure 2, non-magnetic and magnetic forces acting on the micro-UFO during its movement on the *x*-axis in the liquid environment are shown in Figure 2. During the lateral motion of the microrobot, it is subject to a friction force originating from the liquid in which the micro-UFO is located. Due to the hydrodynamic structure of the liquid, friction force is influenced by the direction of micro-UFO motion. This effect creates a phase difference between the lifter magnet and the carrier magnet centers. The reason for this is that the acceleration of the micro-UFO is counteracted by a friction force lower than that of the lifter magnet.

In the micro-UFO levitation system, an acrylic container with DI-water were experimented. Pyrolytic graphite was placed on the surface of the vessel and the micro-UFO was placed above it. 

### 2.2. Theoretical Background

Non-magnetic forces, which are gravitational, buoyancy, and drag forces, are shown in Figure 1, and can be expressed individually as

(1) $$F_{r}=m_{r}g$$

(2) $$F_{B}=V_{r}\left(\rho_{r}-\rho_{f}\right)g$$

(3) $$F_{D}=\frac{1}{2}c_{d}\rho_{f}Av_{\;}\left|v_{\;}\right|$$

In the corner regions of a microrobot, the vortex effect is arisen, although, it is mainly operated in laminar flow with low Reynolds number. For this reason, rather than using the “linear damping term” in Equation (3), the “quadratic viscous term” is preferred to calculate drag force more precisely. Then, attractive magnetic force induced by lifter magnet, $F_{m\;}$, can be expressed in the volume of the suspended magnet [29],

(4) $$F_{m}=m_{z}\frac{\partial B_{z}}{\partial z}\overset{\;}{\underset{\;v}{∭}}dv$$

where $m_{z}$ is the magnetization of the volume element *dv* of the carrier magnet. Alternatively, the differential form is derived by Thomson’s formulation as

(5) $$F_{m}=\frac{V_{p}\chi }{2\mu_{0}\mu_{r}}\left(B\nabla \right)B$$

Boundary conditions, such as $\nabla B^{2}>0$ and $(\mu_{r}-1)<0$, should be satisfied to ensure stability at the equilibrium point. From this equation, a magnetic field gradient is needed so that a linear force can be generated on the micro-UFO. The position of the micro-UFO can be controlled by positioning the magnetic field gradient relative to the micro-UFO. The last magnetic force induced by pyrolytic graphite, that is, the diamagnetic repulsive force on the micro-UFO, can be derived by assuming the material is uniform. In this case, diamagnetic force of per unit volume dv on the magnetic field of the micro-magnet can be expressed as

(6) $$df=M_{\mathrm{dia}}\left(\nabla B\right)dv$$

where $M_{\mathrm{dia}}$ is the magnetization at the location of the diamagnetic material in the magnetic field of the carrier magnet, respectively. Since $\chi_{\mathrm{dia}}$ value is so small, it can be expressed as

(7) $$M_{\mathrm{dia}}=\;\frac{\chi_{\mathrm{dia}}}{\mu_{0}}B$$

The Equation (7) is substituted in to (6), and the volume integral for all the diamagnetic material can be taken. Therefore, the force between the diamagnetic material and the carrier magnet can be obtained as follows:

(8) $$F_{\mathrm{dia},x}=\;\frac{\chi_{\mathrm{dia}}}{2\mu_{0}}\underset{V}{∭}\left(\frac{\partial \left||B\right|{|}^{2}}{\partial x}\right)dv$$

(9) $$F_{\mathrm{dia},y}=\frac{\chi_{\mathrm{dia}}}{2\mu_{0}}\underset{V}{∭}\left(\frac{\partial \left||B\right|{|}^{2}}{\partial y}\right)dv$$

(10) $$F_{\mathrm{dia},z}=\frac{\chi_{\mathrm{dia}}}{2\mu_{0}}\underset{V}{∭}\left(\frac{\partial \left||B\right|{|}^{2}}{\partial z}\right)dv$$

Furthermore, it is possible to simplify the diamagnetic force components in *x*, *y*, *z* directions according to Ostrogradsky’s divergence law [30] as $F_{\mathrm{dia},x},\;F_{\mathrm{dia},y},\;F_{\mathrm{dia},z}$ for ds, which is the surface area unit of the diamagnetic material

(11) $$F_{\mathrm{dia},\;x}=\frac{\chi_{\mathrm{dia}}}{\mu_{0}}\underset{s}{∯}\left||B|\right|{}^{2}n_{x}ds$$

(12) $$F_{\mathrm{dia},\;y}=\frac{\chi_{\mathrm{dia}}}{\mu_{0}}\underset{s}{∯}\left||B|\right|{}^{2}n_{y}ds$$

(13) $$F_{\mathrm{dia},\;z}=\frac{\chi_{\mathrm{dia}}}{\mu_{0}}\underset{s}{∯}\left||B|\right|{}^{2}n_{z}ds$$

where $n_{x}$, $n_{y}$, $n_{z}$ are the surface normal vector component of the diamagnetic material in *x*, *y*, *z* direction, respectively. The diamagnetic force between the pyrolytic graphite and the carrier magnet can be obtained through Equations (11)–(13). After determination of the magnetic forces which are given in the equation set (5), (11)–(13), the dynamic model can be expressed by (14)–(16),

(14) $$\ddot{x}=\frac{1}{2m_{r}}c_{d}\rho_{f}A_{x}v_{x}\left|v_{x}\right|+\frac{\left(F_{m,x}+F_{g,x}\right)}{m_{r}}$$

(15) $$\ddot{y}=\frac{1}{2m_{r}}c_{d}\rho_{f}A_{y}v_{y}\left|v_{y}\right|+\frac{\left(F_{m,y}+F_{g,y}\right)}{m_{r}}$$

(16) $$\ddot{z}=\frac{1}{2m_{r}}c_{d}\rho_{f}A_{z}v_{z}\left|v_{z}\right|+\frac{V_{r}g}{m_{r}}\left(\rho_{r}-\rho_{f}\right)-g+\frac{\left(F_{m,z}+F_{g,z}\right)}{m_{r}}$$

In addition, the sum of the magnetic forces acting on the micro-UFO can be collected into a single expression to simplify as net force in Equation (17),

(17) $$F_{\mathrm{net}}=F_{m}+F_{g}$$

The parameters of micro-UFO and its environment, which are used in both simulation and experimental studies, are given in Table 2 (robot mass, cross sectional areas, and volume are obtained by Solidworks 3D (version 2015, Dassault Systèmes, Vélizy-Villacoublay, France)).

### 2.3. FEM Analysis

**Figure 3:**
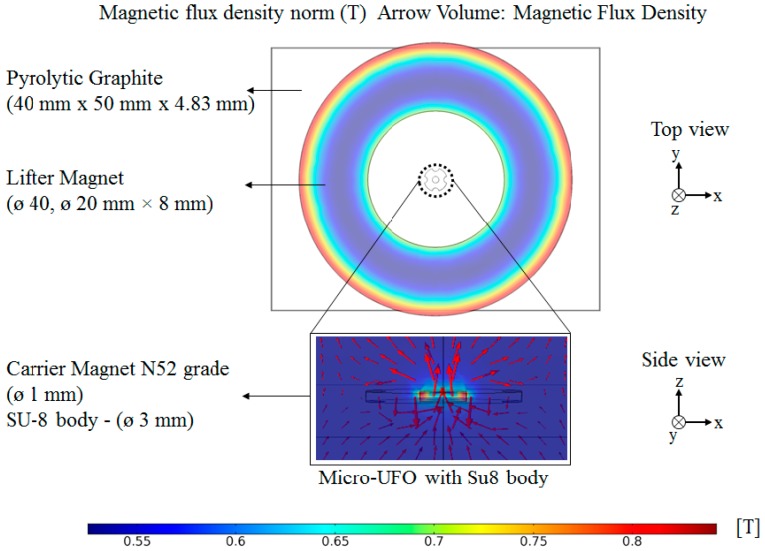
The effect of the magnetic field on the micro-UFO and the measurements of the materials used are shown. As expected in the lifter magnet, the magnetic field is seen with a higher [T] (red color), due to the polarity of the pole lines in the corner regions. In the micro-UFO, the same situation is seen in the magnified image. It has also been shown that the lines are in the *z*-axis direction (side view) since the magnets are placed in N-S-N-S. Used computer: Intel Xeon E4820 * 4 processors (32 core), 128 GB RAM.

According to the values given in Table 2, theoretically, drag force and magnetic force cannot be calculated. Due to complex geometry of the micro-UFO, convergence may not be possible in the integration processes that need to be calculated. There is also no further simplification in the theoretical formulations for the determination of $c_{d}$ and $F_{\mathrm{net}}$ values. Since each force acting on the micro-UFO cannot be calculated analytically, it is necessary to reduce the process load by utilizing numerical methods. For this reason, COMSOL^®^ magnetic field no currents (MFNC) module for magnetic force calculation and COMSOL^®^ fluid structure interface (FIS) module for drag force calculation are applied, to find out the stress and force exerted on the surface of micro-UFO. The micro-UFO has a design of full circle with four half-moon shape pockets. The overall view of the computational fluid dynamics (CFD) model, the materials’ settings and their dimensions are shown in Figure 3.

The calculation of the $c_{d}$ requires that the $F_{d}$ should be determined first. Accordingly, computational fluid dynamics (CFD) must be applied to $F_{d}$ computation of the micro-UFO moving in an enclosed fluid environment. Parametric analysis is necessary in order to calculate more precise drag force coefficient. The analysis has been done in the FIS module in COMSOL^®^. As a result of the analysis, $c_{d}$ calculation has been completed, depending on the external surface stress, including gravitational and hydrodynamic effects of the micro-UFO on *z*-axis. In the analysis, $c_{d}$ calculations were performed by the comparison of two results that are the micro-UFOs levitated in the *z*-axis in the fluid channel and moving mesh on the *x*-axis.

**Figure 4:**
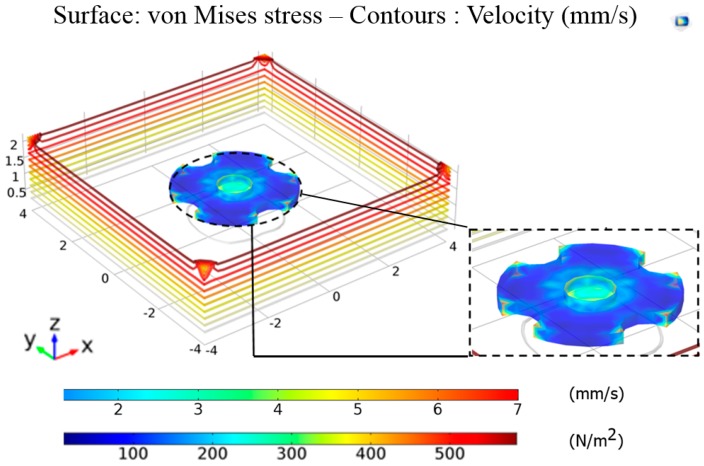
The surface stress plot at the velocity of 5 mm/s. $F_{D}$ is calculated by integrating the external surface stresses generated on the micro-UFO during its movement on the *z*-axis. Velocity values of micro-UFO are in the range of 0–7 mm/s, and represented by the upper legend. According to contour lines on the micro-UFO, the lower legend represents surface stress on the micro-UFO. It can be seen more intensively at corner and intersection points between SU-8 and stabilizer magnet, same as mentioned in Figure 3. The reason is that the different properties of the intersection point of su-8 and carrier magnet hardness affect the homogeneous distribution in stresses. So, the intersection point of su-8 magnet has more stress. Moreover, there is more stress in the micro-UFO corner areas as well. This is due to the high flow rates caused by sharpness of the corner areas.

**Figure 5:**
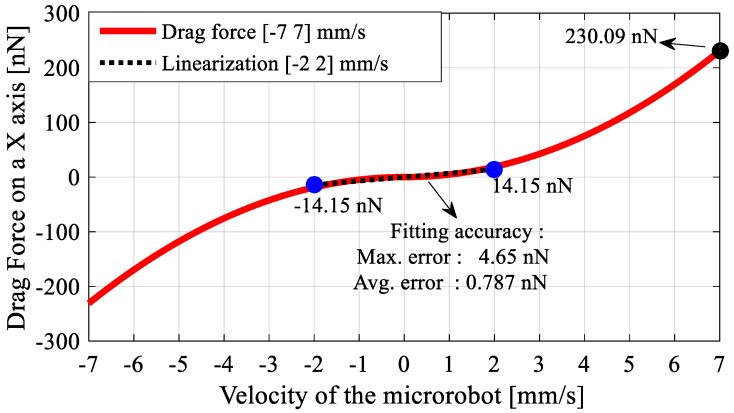
Exhibits a parabolic drag force characteristic depending on the velocity of micro-UFO. The drag force change is less for the [−2, 2] mm/s range, which is the microrobot motion speed. For this reason, the system can be expressed by linear equation, by performing curve fitting at the specified interval. For the equation proposed as $F_{d}=kV_{r}$, a constant coefficient was found to be $k=7.075\times 10^{-9}\;\mathrm{kg}/s^{2}$. Due to the symmetric structure of the microrobot, the force calculation is preferably shown only for *x*-axis.

The change in $F_{D}$ with respect to micro-UFO’s velocity, that is obtained from the analysis shown in Figure 4, is given in Figure 5. Positive and negative expressions for the micro-UFO moving at [−2 2] mm/s represent the direction of movement.

**Figure 6:**
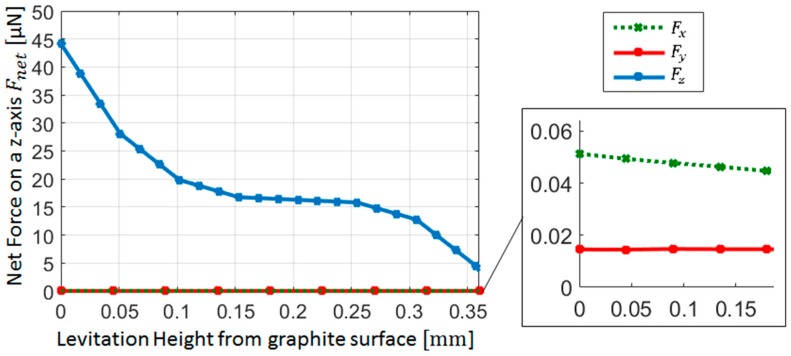
The net magnetic force characteristics acting on the micro-UFO for each one of the three axes vs to levitation distance. It has been shown that the *x*- and *y*-axis forces are in the 1/10^3^ ratio according to the force value generated in the *z*-axis. However, when the acting forces on the *x*- and *y*-axes of the micro-UFO are small, 40–50 nN, the motion that occurs can be due to *x*, *y* forces being bigger than the drag force (Figure 5).

**Figure 7:**
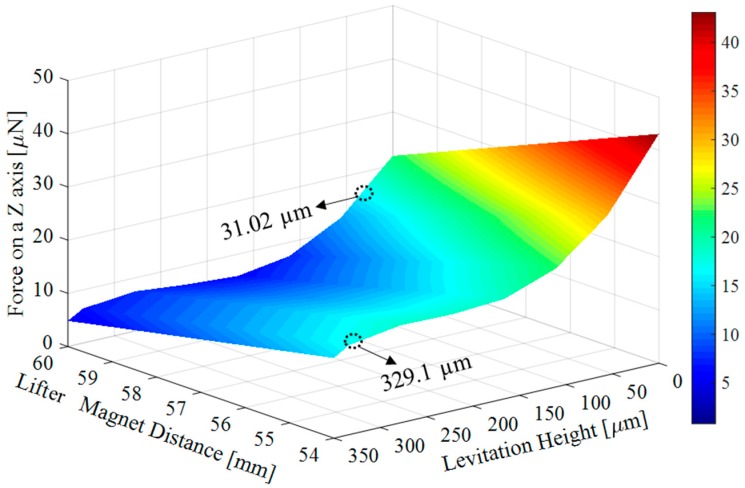
Shows the effect of the forces exerted on the micro-UFO depending on the distance between the “Lifter Magnet” and the “Pyrolytic Graphite”. Accordingly, as the distance increases, the total forces on *z*-axis decreases exponentially. Minimum levitation point when lifter magnet distance is 60 mm, 31.02 µm; maximum levitation point is 54 mm, 329.1 µm are calculated.

Now, only the magnetic and diamagnetic forces are left to calculate. For this reason, as a second step after drag force calculation, the net magnetic force, $F_{\mathrm{net}}$, shall be calculated. $F_{\mathrm{net}}$ varies with the levitation height of the lifter magnet from pyrolytic graphite. The distance between micro-UFO and pyrolytic graphite, when the forces acting on the micro-UFO are equal to zero, represents the levitation height. Analysis results of the levitation distance versus the net magnetic force is given in Figure 6. Accordingly, the lifter magnet position effect on levitation characteristic is demonstrated in Figure 7. The analysis of the experimental setup was solved using the COMSOL^®^ direct solver method to obtain more accurate results.

### 2.4. FEM Analysis of Levitation Characteristic’s Result

The result of the analysis is calculated by using both the Maxwell stress tensor and global matrix evaluation methods, and it is seen that the results are the same. Lifter magnet is far away from the micro-UFO, so the magnetic field force lines can occur symmetrically. Since the effective length of the lifter magnet is larger than the physical length by an amount which is roughly equal to the air gap, the forces generated in the *x*- and *y*-axes are weaker than the *z*-axis. During levitation on the *z*-axis, net magnetic forces generated are in the order of micrometers, while in *x*- and *y*-axes, the forces falling in the nano order are about 1/$10^{3}$. Figure 6 shows the net magnetic force values calculated when the phase difference is 0.1 mm and the pyrolytic graphite-lifter magnet distance is about 54 mm.

Along with the net magnetic force, drag force, buoyant force, and gravitational force are acting on the micro-UFO at the same time. Thus, they can be summed as $F_{T}$ (total force), as it is shown in Equation (18).

(18) $$F_{T}=F_{\mathrm{net},z}-F_{D,z}+F_{B,z}-F_{r,z}$$

In addition, $F_{D}=14.15\;\mathrm{nN}$ is calculated when the micro-UFO velocity is about 2 mm/s. So, the required magnetic force $F_{\mathrm{net},z}$ for the levitation is calculated as 15.967 μN. The corresponding force value given in Figure 6 is shown as marked on the surface graph. As a result, the robot is separated from the stabilization point at a levitation height of 333.8 μm, according to the analysis. In addition, there is a flat profile with a linear slope at the levitation height: 100–300 µm. The area outside the region concerned may be called the region of instability; within this region, the micro-UFO can be stably operated. The total force obtained on the *z*-axis is shown as surface plot in Figure 7.

The maximum levitation height relative to pyrolytic graphite is shown in Figure 7. According to this, when *z*-axis distance between the “lifter magnet” and the “pyrolytic graphite” is more than 54 mm, micro-UFO reaches instability, and detaches from suspension at a height of 329.1 µm. The reason is that the required magnetic force $F_{\mathrm{net},z}$, which is calculated as 15.967 μN, cannot be obtained after 54 mm.

## 3. Materials and Methods

### 3.1. Permanent Magnet Surface Coating

**Figure 8:**
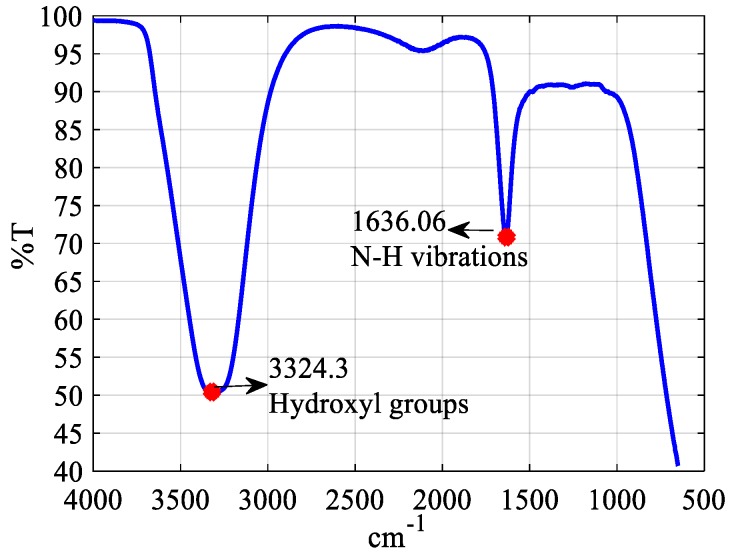
Fourier transform infrared (FTIR) spectrum of magnet coated with polydopamine (PDA). The peak between wavelength of 1500–1650 cm^−1^ indicates N–H vibrations, and peak between wavelength of 3200–3600 cm^−1^ indicates hydroxyl groups in the structure. This proves successful coating of magnet surface.

Since most of the micro-UFO’s target application areas are biomedical applications, the surface of the micro-UFO is coated with polydopamine (PDA), which is a convenient material for biomedical applications [31]. Accordingly, after adding 100 mg of dopamine powder (Merck & Company, Inc., Kenilworth, NJ, USA) in 50 mL of purified water, Tris powder (100 mg) was dissolved in the prepared mixture. Starting with the beginning of the coating, the color of the solution gradually gets darker. In the end of the coating, the magnet coated with the PDA is separated from the solution using another magnet. After coating of magnet, Fourier transform infrared (FTIR) measurements were applied by Perkin Elmer Spectrum 100 (PerkinElmer, Inc., Waltham, MA, USA), which contains diamond crystals for reflection. After placing coated magnet in accessory, peaks of spectrum were measured at the wavelength of 1500–1650 cm^−1^ and 3200–3600 cm^−1^; these peaks indicate atomic vibrations of the hydroxyl groups and N–H vibrations in the structure (Figure 8). The presence of coating layer is confirmed by the FTIR spectrum observed in Figure 7. This demonstrates that the permanent magnet used in the robot’s body is successfully coated with PDA for biomedical applications.

### 3.2. Micro-UFO Fabrication

**Figure 9:**
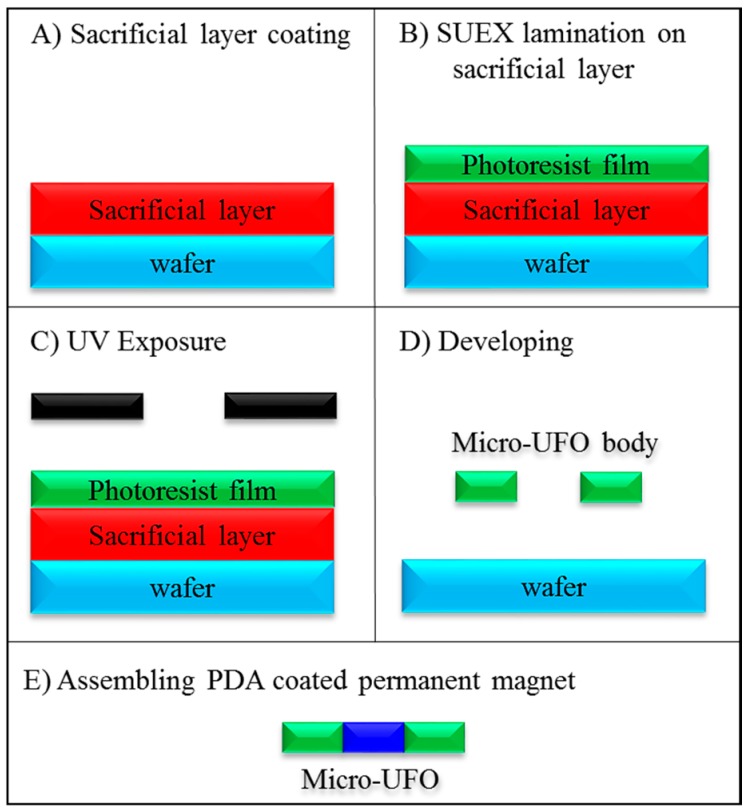
Fabrication process of the micro-UFO. (**A**) Sacrificial layer coating; (**B**) SUEX lamination on sacrificial layer; (**C**) UV Exposure; (**D**) Developing; (**E**) Assembling polydopamine (PDA) coated permanent magnet.

Micro-UFO manufacturing steps are shown in Figure 9. AZ 1505, a positive photoresist is firstly coated onto a substrate surface as a sacrificial layer (MicroChemicals GmbH, Ulm, Germany). After that, a negative adhesive film with a thickness of 200 microns was laminated on sacrificial layer by rolling at 90 °C on hot plate (SUEX 200, DJ MicroLaminates, Inc., Sudbury, MA, USA). The exposure process on film is applied and then final polymer micro-UFO body was obtained by the pattern development process. As the last step, neodymium (Nd_2_Fe_14_B) circular permanent magnet (N-52 grade) with the dimensions of 1 mm × 0.25 mm (diameter-thickness) is coated with PDA. Then PDA-coated biocompatible permanent magnet was assembled in micro-UFO body.

## 4. Experimental Setup and Test Results

### 4.1. Experimental Setup

**Figure 10:**
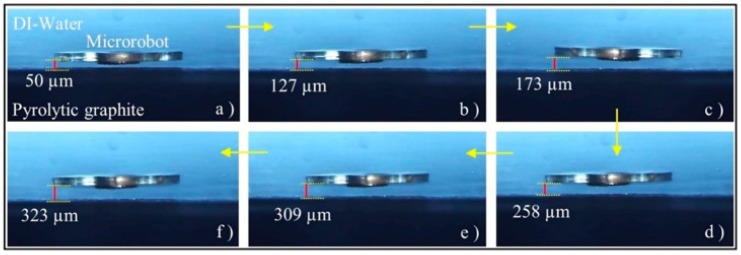
The experimental results show the micro-UFO with different levitation height depending on the distance between “Lifter Magnet” and the “Pyrolytic Graphite (PG)”. The experiment was recorded by acquiring the position data with the laser distance sensor in each movement step. The micro-UFO has been shown to operate at a maximum stabilization level of 333.8 μm in the current system. In figure, the micro robot is shown at various levitation heights based on lifter-magnet position relative to PG; (**a**) 50 μm, (**b**) 127 μm, (**c**) 173 μm, (**d**) 258 μm, (**e**) 309 μm and (**f**) 323 μm.

The whole experimental setup where the levitation and motion characterization experiments of the micro-UFO are performed is already shown in Figure 2. The micro-UFO is levitated by the movement of the lifter magnet in the order of microns along the *z*-axis. It exhibits a levitation characteristic depending on the magnets and properties used within the system. The reason for using a ring-shaped lifter magnet is that it has a hollow in its structure, which is required for laser measurement. Because of the gap in the middle of the ring-shaped structure (20 mm diameter), the laser is aligned with the micro-UFO in the vertical position. The PI Stage, which we controlled through a computer interface, was moved by 50 μm on the *z*-axis, followed by 10 μm steps as the first levitation occurred. When the micro-UFO is in the levitation state, the force behavior of the lifter magnet in the motion range of about 6 mm is observed. The measurements made in terms of distance between different levitation heights and the micro-UFO positions are given in Figure 10.

**Figure 11:**
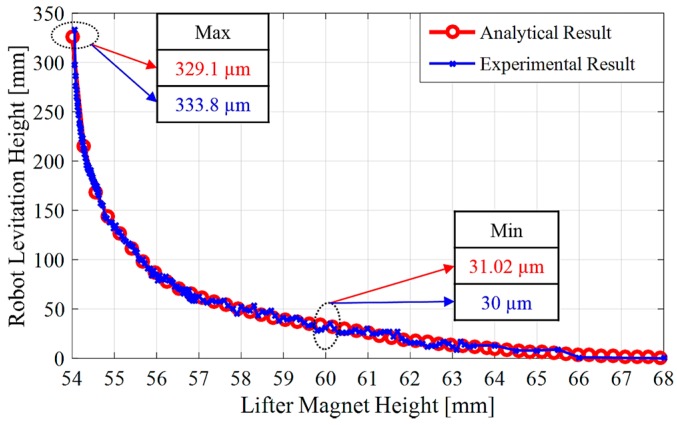
Finite element method (FEM) analysis and experimentally compared levitation heights according to lifter magnet height. Accordingly, it is seen that the obtained levitation characteristics are very close to each other.

In addition, the change in the 6 mm between the lifter magnet and the pyrolytic graphite also affects the levitation height of the micro-UFO, as shown in Figure 11.

Considering the objects and their motion field to be manipulated by the micro-UFO in lab-on-a-chip applications, the maximum of 333.8 μm and minimum of 30 μm levitation height obtained from the experimental results are sufficient. The diamagnetic levitation method we developed, may vary according to various system configurations to be applied. The proposed manipulation method allows this micro-UFO design and driving magnet properties to be modified to achieve specific levitation intervals for different anticipations.

### 4.2. Accurate Positioning and Phase Difference Characterization

**Figure 12:**
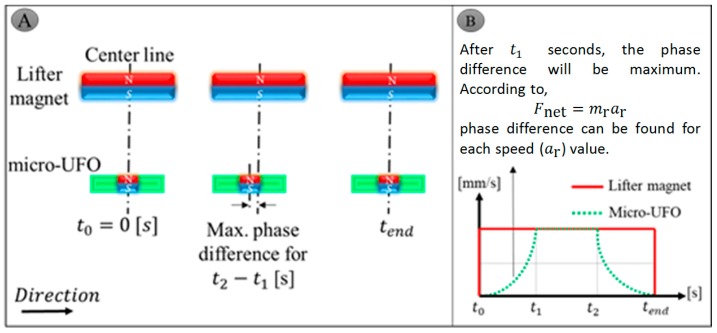
The phase difference between micro-UFO and lifter magnet starts from $\;t_{0}$ starting moment is shown in (**A**). After a period of time $t_{1}$ (**B**), the speed with the carrier magnet will be equal, and the phase difference reaching the maximum will begin to decrease after this point. Figure 6 and Table 2, micro-UFO acceleration, $a_{r}$, can be calculated. For this micro-UFO and lifter magnet with velocity profile and acceleration specified, the difference phase difference can be expressed easily.

The lifter magnet attached to the micro-stages is moving in the air. For this reason, the stage speed is equal to the speed of the lifter magnet, and has a constant speed profile. The micro-UFO is placed in the fluid, and its velocity increases until it reaches the speed of the lifter magnet. In this case, it creates a phase difference. The micro-UFO, which reaches the movement at the same speed as the lifter magnet, then goes on constantly, and is reset to zero by descending, when the lifter magnet movement is completed. The corresponding phase difference is shown in Figure 12A, and the motion profile is shown in Figure 12B. With the parameter values of the magnetic force, it is possible to calculate the phase difference approximately theoretically.

**Figure 13:**
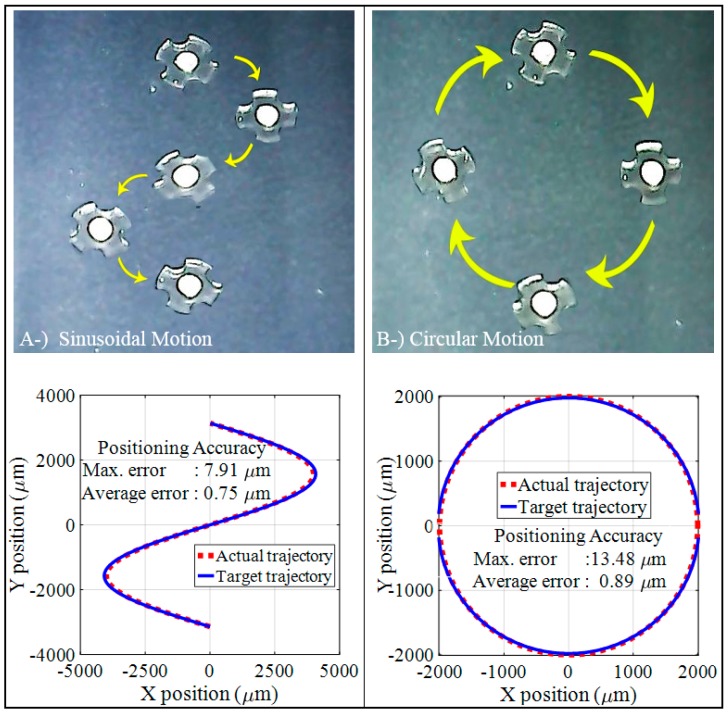
The sinusoidal and circular orbit trajectories have been successfully tracked. (**A**) shows the sinusoidal trajectory followed by the signal input given as amplitude 4 mm. In (**B**), a circular orbit with a radius of 2 mm was followed.

The movement of the micro-UFO in an untethered manner is observed in the *x*- and *y*-axes at a constant levitation height. Accordingly, the computer control interface has been coded for sinusoidal, circle motion orbitals, in which necessary variables can be adjusted and experimentally applied in Figure 13. In this figure, the maximum 7.91 µm for sinusoidal, and 13.48 µm for circular, and average errors 0.75 µm for sinusoidal and 0.89 µm for circular of the micro-UFO, are given for a high speed as 2 mm/s. It has been observed that the maximum error level increases when the trajectory has sudden turn and sharp movements. 

**Figure 14:**
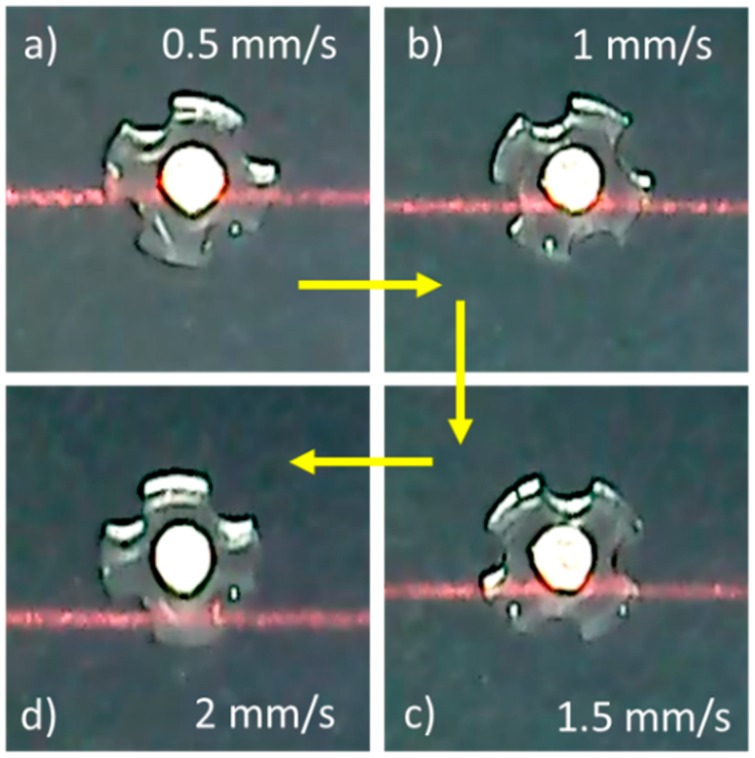
Depending on change of the micro-stage speed: (**a**) 0.5 mm/s; (**b**) 1 mm/s; (**c**) 1.5 mm/s; (**d**) 2 mm/s, micro-UFO delay relative to different lifter magnet speeds are shown. The red line belongs to the laser which is sent from the center of the lifter magnet.

As a result of experimental studies and the observations made, the micro-UFO has the ability to precisely move and position in the proposed system. Micro-UFO performed smooth motion at the desired speeds, ending its movement at the point where it would be coincident with the lifter magnet, as expected. Thus, the micro-UFO with diamagnetic levitation proved to have a precise positioning capability. Due to the fact that the micro-UFO is levitated in an untethered manner, it is exposed to fluid-induced frictional resistance in the fluid. Besides, it has an inertia originating from its own mass. Because of all these factors, the micro-UFO follows the motion trajectory in the *x*- and *y*-axes with a delay in relation with the ring magnitude, i.e., the robot starts its motion in a misaligned manner (Figure 14). The offset (delay) from the center of the lifter magnet is defined as “phase difference”.

**Figure 15:**
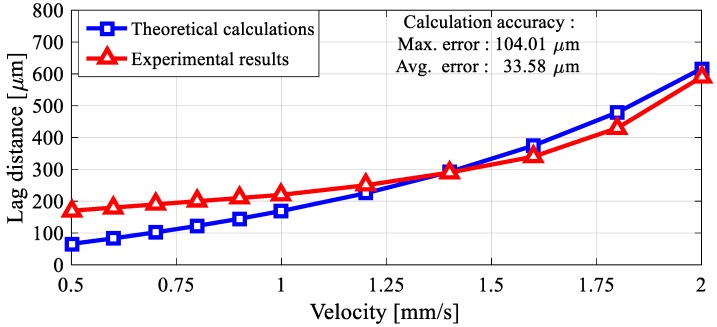
At higher velocities of about 1.4 mm/s, the phase difference is characterized by an exponential increasing trend, and at lower speeds with linearized phase difference characterization, as expected. It is obvious that the phase difference falls below 150 μm as the same linear change is likely to continue even at small speeds of 0.5 mm/s. This is an indication that non-delayed positioning can be performed by neglecting the phase difference at the speed requirements of the nanometer range, which will be needed especially in lab-on-a-chip applications.

The phase difference is a parameter that is controlled by the computer interface, and depends on the speed of the moving ring-shape lifter magnet on the micro stage. As the speed of progression increases, the phase difference increases as a result of experiments. The fact that the phase differences are on the move do not cause a situation to hinder precise speed and position control. The micro-UFO follows the same trajectory as the lifter magnet on *x*- and *y*-axes. After the motion is finished, the micro-UFO ends its movement in the concentric state with the lifter magnet. The amount of delay due to the micro-stage speed is experimentally determined, and shown in Figure 15.

### 4.3. Head Bending Reaction

**Figure 16:**
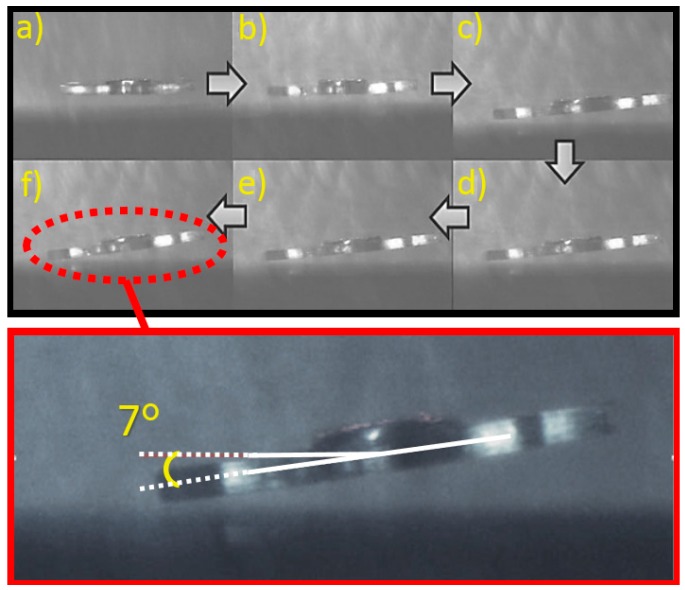
The cause of the head-tilt reaction is to suspend the directional changes of the vector field forces acting on the micro-UFO. The head-tilt angle is a parameter proportional to the phase difference. The increase in phase difference also causes an increase in head-tilt angle. As can be seen in the figure, in the experimental studies performed, the head-tilt angle of 7° is displayed. The increase on gradual tilting of the microrobot can be seen in the figure from (**a**) to (**f**).

The resulting phase difference causes vectorial directional changes in the resultant magnetic field forces acting on the micro-UFO. As a result of observations we made during experimental studies, it was also seen that the micro-UFO performed a head-tilting motion in the direction of movement in the case of phase difference occurrence (Figure 16).

## 5. Discussion and Conclusions

### 5.1. General

In this system, which we designed with our team, we offer an innovative micro-UFO working mechanism suspended by diamagnetic levitation, unlike other studies. In the system we designed, the micro-UFO was able to successfully track the maximum range of motion of the linear stage at 500 nm through the lifter magnet after it was suspended at certain heights. With the use of nano-positioner with higher motion sensitivity, it is obvious that higher resolution position capabilities can be achieved successfully. Another innovative feature of the designed micro-UFO, which is different from micro-UFOs providing permanent magnet motion, is that it has a more suitable design for biomedical applications. PDA coated micro-UFO also have the ability to prevent contamination.

### 5.2. Levitation on z-Axis

It is possible to control the movement of *x*-*y* and *z*-axes within the microfluidic channel of the micro-UFO. By means of finite element analysis in COMSOL, the levitation height limits of the micro-UFO were successfully determined and compared with the experimental results. Magnetic analysis showed that the micro-UFO showed linear surface behavior in the range of 90–280 μm according to surface force graph on *z*-axis direction. Experimentally, it was determined that the current micro-UFO levitation characteristic has an unstable structure in the range of 0 to 30 microns, and 290 to 333.8 microns, and the stable working range of the system is in the range of 30 to 290 microns. Accordingly, when compared with the experimental results, the analysis results were found to be within the experimental limits. If the analytical findings obtained are used on the system, it has been proved that safe and linear behavior range can be studied.

### 5.3. Drag Force—Lifter Magnet Effects

The distance between the lifter magnet and the pyrolytic graphite, as well as the range of levitation, are calculated numerically. Compared to the experimental results, it has been shown that the working range of 54–58 mm is common with current experimental setup. Although the micro-UFO can be controlled beyond this range, it has been shown that the linear zone is the better working range. As shown in the experimental results, the numerical analysis result showed that the micro-UFO in the determined range has more stable characteristics. The result of the drag force analysis shows that the viscous effect of the water is not much higher than micro-UFO. It is calculated that the maximum value of drag force is about 5% of the force required to levitate the robot. If micro-stages’ operating speed range is set to ±2 (mm/s), it is estimated that the drag force will be less than 0.75%. Thus, we can say that the drag force can be neglected, and the system can be linearized by neglecting the drag force effect. For future studies, a linear control technique can be potentially utilized in this application.

### 5.4. Center Alignment

Precise position control is also provided on the *x*- and *y*-axes of the micro-UFO in the direction of the experimental data. The mobility of the micro-UFO has been tested in motion trajectories that may be encountered in different scenarios and work areas. The micro-UFO has successfully followed the sinusoidal and circular orbits, in addition to the linear motion. As you can see in COMSOL analysis, when 0.1 mm phase difference occurs between lifter-carrier magnets’ center, it is calculated that forces generated in *x*- and *y*-axes are about 1/10^3^ of the force in *z*-axis. Therefore, predominant *z*-axis force cannot be affected by *x*- and *y*-axes forces dramatically, and levitation height does not change during motion of the micro-UFO. Furthermore, it is always centralized with lifter magnet poles that provide stable trajectory tracking.

### 5.5. Phase Difference

According to levitation characteristics with the help of motorized micro-stage motions, the phase difference during the movements is seen as a factor that will force us to do real-time position control. On the other hand, for many biomedical applications, nanometric speeds are being used. In this case, the phase difference was found to be negligible as a result of our experimental studies. According to the analysis and experimental results regarding the phase difference, the torque and force values applied on the robot were not affected. The micro-stage is moving at a maximum speed of 2 mm/s. In this case, an offset of 0.6 mm is calculated and shown in Figure 15. It can be seen that the calculated phase difference is very small (1/66) compared to the lifter magnet size. Thus, it is observed that there will be no effect on the magnetic force and torque produced. In addition, when the phase difference is high, the head-tilting reaction observed at high speed levels is likewise observed to be negligible within the operating speed requirements of the applications. However, the possibility of giving capillary damage to the surface boundaries of the head-tilt reaction cannot be overlooked, especially in low run-up working conditions. In this regard, in future studies, closed loop control-based studies will be carried out to prevent the head-tilt reaction.

## Figures and Tables

**Table 1:** System Model Parameters.

Symbol	Quantity	Units
$F_{B}$	Buoyant force	N
$F_{g}$	Diamagnetic force	N
$F_{D}$	Drag force	N
$F_{m}$	Magnetic force	N
$F_{r}$	Gravitational force	N
$T_{x}$	Unwanted torque	Nm
B	Magnetic flux density	T
H	Magnetic field strength	A/m
M	Magnetization vector	A/m
χ	Magnetic insulation coefficient	-
$\mu_{r}$	Relative permeability	-
$V_{p}$	Volume of a particle	$m^{3}$
$m_{r}$	Robot mass	kg
g	Gravitational acceleration	$m/s^{2}$
$V_{r}$	Robot volume	$m^{3}$
$\rho_{f}$	Fluid density	$\mathrm{kg}/m^{3}$
$\rho_{r}$	Robot density	$\mathrm{kg}/m^{3}$
$a_{r}$	Robot acceleration	$\mathrm{mm}/s^{2}$

**Table 2:** Micro-untethered floating object (Micro-UFO) properties and nonmagnetic forces.

Symbol	Quantity	Units
$m_{r}$	2.929751 × $10^{-6}$	kg
g	9.81	m/$s^{2}$
$\rho_{r}$	1798.374	kg/$m^{3}$
$\rho_{f}$	998.2071	kg/$m^{3}$
$A_{x}$	1.229066 × $10^{-6}$	$m^{2}$
$A_{y}$	1.229066 × $10^{-6}$	$m^{2}$
$A_{z}$	8.15402 × $10^{-6}$	$m^{2}$
$V_{r}$	1.61911 × $10^{-9}$	$m^{3}$
*I*	23.62 × $10^{-12}$	kg·$m^{2}$
$F_{B,z}$	12.788	μN
$F_{r,z}$	28.741	μN
